# Emergence and spread of SARS-CoV-2 variants of concern in Canada: a retrospective analysis from clinical and wastewater data

**DOI:** 10.1186/s12879-024-08997-8

**Published:** 2024-01-29

**Authors:** David Champredon, Devan Becker, Shelley W. Peterson, Edgard Mejia, Nikho Hizon, Andrea Schertzer, Mohamed Djebli, Femi F. Oloye, Yuwei Xie, Mohsen Asadi, Jenna Cantin, Xia Pu, Charles A. Osunla, Markus Brinkmann, Kerry N. McPhedran, Mark R. Servos, John P. Giesy, Chand Mangat

**Affiliations:** 1 Public Health Agency of Canada, National Microbiology Laboratory, Public Health Risk Sciences Division, Guelph, ON Canada; 2 Public Health Agency of Canada, National Microbiology Laboratory, One Health Division, Winnipeg, MB Canada; 3 Public Health Agency of Canada, Centre for Immunization and Respiratory Infectious Diseases, Ottawa, ON Canada; 4 Toxicology Program, University of Saskatchewan, Saskatoon, SK Canada; 5 Department of Chemistry, Division of Physical and Computational Sciences, University of Pittsburgh at Bradford, Bradford, United States; 6 Department of Civil, Geological and Environmental Engineering, College of Engineering, University of Saskatchewan, Saskatoon, SK Canada; 7 School of Environment and Sustainability, University of Saskatchewan, Saskatoon, SK Canada; 8 Global Institute for Water Security, University of Saskatchewan, Saskatoon, SK Canada; 9 Department of Biology, University of Waterloo, Waterloo, ON Canada; 10 Department of Veterinary Biomedical Sciences, University of Saskatchewan, Saskatoon, SK Canada; 11 Department of Environmental Sciences, Baylor University, Waco, TX USA; 12 Department of Zoology and Center for Integrative Toxicology, Michigan State University, East Lansing, MI USA

**Keywords:** SARS-CoV-2lineages, Molecular analysis, Lineage identification, Variant of concern, Clinical samples: Omicron, Wastewater-based epidemiology

## Abstract

**Background:**

The spread of SARS-CoV-2 has been studied at unprecedented levels worldwide. In jurisdictions where molecular analysis was performed on large scales, the emergence and competition of numerous SARS-CoV-2lineages have been observed in near real-time. Lineage identification, traditionally performed from clinical samples, can also be determined by sampling wastewater from sewersheds serving populations of interest. Variants of concern (VOCs) and SARS-CoV-2 lineages associated with increased transmissibility and/or severity are of particular interest.

**Method:**

Here, we consider clinical and wastewater data sources to assess the emergence and spread of VOCs in Canada retrospectively.

**Results:**

We show that, overall, wastewater-based VOC identification provides similar insights to the surveillance based on clinical samples. Based on clinical data, we observed synchrony in VOC introduction as well as similar emergence speeds across most Canadian provinces despite the large geographical size of the country and differences in provincial public health measures.

**Conclusion:**

In particular, it took approximately four months for VOC Alpha and Delta to contribute to half of the incidence. In contrast, VOC Omicron achieved the same contribution in less than one month. This study provides significant benchmarks to enhance planning for future VOCs, and to some extent for future pandemics caused by other pathogens, by quantifying the rate of SARS-CoV-2 VOCs invasion in Canada.

**Supplementary Information:**

The online version contains supplementary material available at 10.1186/s12879-024-08997-8.

## Introduction

Unprecedented clinical, genomic, and environmental surveillance applied to manage the on-going coronavirus disease 2019 (COVID-19) pandemic has allowed the scientific community to observe the emergence and spread of multiple lineages of severe acute respiratory syndrome coronavirus 2 (SARS-CoV-2). A consequence of the global prevalence of COVID-19 has been the emergence of SARS-CoV-2 variants, which have been closely monitored due to the acquisition of mutations that confer increased transmissibility or greater evasion of immune response functions [1–4]. Variants associated with particularly high transmission and/or severity are designated “Variants of Concern” (VOC) by the World Health Organization and some VOCs have managed to propagate rapidly on a global scale. The first prominent and global VOC emerging in early 2021 was Alpha (lineage B.1.1.7), followed in June 2021 by Delta (lineage B.1.617.2), and since December 2021 Omicron (lineage B.1.1.529) has rapidly become the predominant strain. Some VOCs have not become dominant on a global scale, but have nevertheless reached a significant frequency at least regionally including Gamma in Brazil [5] and Beta in South Africa [2].

In addition to the traditional surveillance from clinical samples, wastewater-based epidemiology (WBE) can determine the proportion of VOC RNA in a given sewershed by use of sequencing or polymerase chain reaction (PCR) techniques [6–8]. Despite the inherent uncertainties in measuring viral concentration or estimating variants diversity in wastewater, WBE has been useful for informing public health officials and the general public on the status and trends of outbreaks [9, 10]. Hence, it is possible to use both data sources, clinical and wastewater, to potentially improve confidence in estimates of abundances of VOCs circulating in a given population. Indeed, multiple international studies have shown that the relative abundance of a given VOC in wastewater correlates with the proportion of cases infected with this VOC identified by clinical surveillance [11–20].

In light of the multiple SARS-CoV-2 lineages, a retrospective analysis of the dynamics of emergence and spread by VOCs, using both wastewater and clinical data, can help inform preparedness planning for emergence of other VOCs in the future. Regional and national retrospective studies have been conducted to compare WBE and clinical data during the COVID pandemic [21]. As jurisdictions world-wide are transitioning to management of SARS-CoV-2 as an endemic virus, with accompanying reductions in health surveillance, a clear understanding on how an emerging VOC spreads at a regional and national scale will be useful for informing effective public health policy. However, currently there is a lack of retrospective analysis on a national scale in Canada. Therefore, this study was conducted to retrospectively review the invasion dynamics of the prevalent SARS-CoV-2VOCs that have circulated in Canada, with the timing of emergence and speed of spread observed for those VOCs in several Canadian jurisdictions being quantified and described. Our analysis of VOCs circulation in Canada considers both clinical and wastewater data from VOC Alpha (the first to appear in Canada) up to the emergence of VOC Omicron (early 2022). The objectives of this study were to: (1) evaluate the correlation of VOC dynamic trends between clinical and WBE-based data; (2) assess the ability of clinical and WBE-based data to infer the speed of VOC proportion replacing in different provinces in Canada; (3) explore/discuss the suitability of integrating clinical and WBE approaches to support comparative estimates of infection disease in the future.

## Methods

### Clinical data

The Public Health Agency of Canada (PHAC) compiles a national list of COVID cases that are reported by Canadian Provinces and Territories. For some records in this list, the variant or lineage associated with each infection is identified. The lineage is either confirmed from whole genome sequencing or from “screening” via a discriminatory PCR assay that assesses the presence of variants by targeting specific mutations that are likely variant-defining, given the prior knowledge of circulating lineages nationally. In the event of both results being present, sequencing was prioritized over screening. These sequencing and screening efforts varied during the pandemic and across jurisdictions. In this study, this retrospective assessment focused on the four VOCs that reached non-negligible status in Canada: Alpha (lineage B.1.1.7), Gamma (lineage P.1), Delta (lineage B.1.617.2) and Omicron (lineage B.1.1.529). Thus, all other VOCs (e.g., Beta, Epsilon, Kappa) were ignored here. Because there could be some overlap with the mutations defining variants (e.g., Omicron and Alpha), we applied cut-off dates such that variants were assumed to exist only after those dates: 2020–11-01 for Alpha; 2021–02-01 for Gamma; 2021–03-01 for Delta; 2021–11-15 for Omicron.

### Wastewater data

In October 2020, PHAC, the National Microbiology Laboratory (NML) and Statistics Canada started a pilot program that tested for the presence of SARS-CoV-2in wastewater collected between two to three times per week from 15 wastewater treatment plants in various cities in Canada including Vancouver, Edmonton, Toronto, Montreal, and Halifax. In addition, wastewater was sampled from the three treatment plants in Winnipeg five times a week through a provincial/municipal collaboration. All wastewater samples from Winnipeg, Vancouver, Edmonton, Toronto, Montreal and Halifax were shipped to NML in Winnipeg for qPCR analysis. To expand the population coverage of the NML/Statistic Canada program, qPCR analysis of the wastewater sampled from Saskatoon performed by the University of Saskatchewan (not NML).

Thus, the wastewater data sampled the population of one large municipality in each province, whereas our clinical data sampled the whole province. The comparison of the proportion of VOCs between clinical and wastewater data is still relevant because the catchment areas of the municipal wastewater treatment plants represent a substantial proportion of its provincial population. The population present in the catchment area of the wastewater treatment plants in Vancouver represents 48% of the total population of British Columbia; 24% of the province of Alberta for Edmonton; 23% of the province of Saskatchewan for Saskatoon; 54% of the province of Manitoba for Winnipeg; 20% of the province of Ontario for Toronto; 20% of the province of Quebec for Montreal; and 46% of the province of Nova Scotia for Halifax. In all sites, the proportion of the circulating VOCs present in a wastewater sample was quantified (i.e., not just a presence/absence test) by two laboratories only, the NML and the University of Saskatchewan.

Briefly, raw-post grit primary influent was shipped on cold-packs for testing to the NML (Winnipeg, Manitoba). Samples were stored at 4 °C and tested within 1–3 days of receipt. In NML, wastewater samples were mixed vigorously and a 30 mL aliquot was centrifuged (4000 g, 4 °C, 20 min) in a swinging bucket centrifuge. The pellet was then resuspended in Buffer RLT (Qiagen) containing 1% 2-mercaptoethanol and subjected to bead beating using a Bead Mill Homogenizer. The samples were then centrifuged (4000 g, 4 °C, 3 min) and total nucleic acids were extracted from the supernatant by use of the Roche MP96 instrument using the Magna Pure 96 DNA and Viral NA Large Volume Kit (Roche Diagnostics, Laval, QC) according to the Plasma External Lysis 4.0 protocol. See [22] for full methodological details. Base viral loads were determined by use of US-CDC N1/N2 assays [23]. The Alpha and Delta lineage assays were performed as previously described in [22]. The Omicron assay is described in Supplementary Material.

Raw influent samples from Saskatoon were processed as previously described in [10, 17] with modifications. Virus in 70 mL whole raw influent was enriched by PEG-8000 precipitation. Total RNA was extracted by use of RNeasy PowerMicrobiome Kit (Qiagen, Ontario, Canada). Percentage of Alpha VOC was determined by N D3L assay [24], while percentages of Delta and Omicron were determined by N200 assay [25]. There were no structured comparisons of laboratory performance between the NML (that analyzed all samples, except the ones from Saskatoon) and the laboratory at the University of Saskatchewan (that analyzed samples from Saskatoon only). However, the latter performed a comparison of concentration measurements between raw influent (the sample type used for Saskatoon in our study) and post grit primary influent samples (the sample type used by NML in our study), all collected in Saskatoon, and found a significant correlation (R^2^ = 0.57, *p* < 0.001, personal communication by Oloye et al.). Moreover, the respective methods of each laboratory remained the same during the study period which allows us to perform the longitudinal analysis presented here, despite potential differences between the two laboratory assays.

### Statistical analysis

Using the PHAC line list, clinical cases that had been identified as one of the four VOCs of interest (through screening or sequencing) were retained. Frequencies of circulation of variant v at time t is therefore defined as p(v, t) = nv(t)/n(t) where nv(t) is the number of clinical cases identified with variant v on day t, n(t) is the number of clinical cases where any variant was identified on day t. A multinomial distribution for nv was assumed. Confidence intervals for proportions of each VOC were simultaneously estimated by use of the function MultinomCI from the R [26] package DescTools version 0.99.44 [27]. To evaluate growth rates (i.e., speed of initial spread) of clinical data during each wave of a given VOC in a given jurisdiction, it was assumed the time-dependent proportions of VOCs followed a logistic growth model. The three parameters defining the logistic function location, steepness and asymptotic value – were estimated from clinical data only. The model was fit independently for each province (using only clinical data) with Markov chain Monte-Carlo implemented in the R library rjags version 4–12 [28]. To calculate the time since introduction in the logistic growth model, a single date of introduction nationwide for each VOC: 2020–11-01 for Alpha, 2021–02-01 for Gamma, 2021–03-01 for Delta and 2021–11-15 for Omicron was assumed. Moreover, to aggregate provincial clinical data into national estimates, a similar logistical growth model was applied, but with a hierarchical structure. A full description of the statistical models is given in the Supplementary Materials.

The VOC proportions estimated from wastewater are simply the variant allele percentages detected by the corresponding assay. No confidence intervals were derived. Moreover, wastewater data was not used to fit the logistic models due to insufficient data at the beginnings of most waves of SARS-CoV-2associated with various VOCs. Hence, growth rate of each VOC were assessed using clinical data only. However, the wastewater-based VOC proportions are compared to the clinical-based ones to assess their similarity.

## Results

Findings are reported for seven Canadian provinces that had a sufficiently large sample size of VOC identified to provide interpretable results: British Columbia (B.C.), Alberta (Alta.), Saskatchewan (Sask.), Manitoba (Man.), Ontario (Ont.), Quebec (Que.) and, Newfoundland and Labrador (N.L.).

### Comparison of VOC dynamic trends between clinical and wastewater-based data

**Fig. 1:**
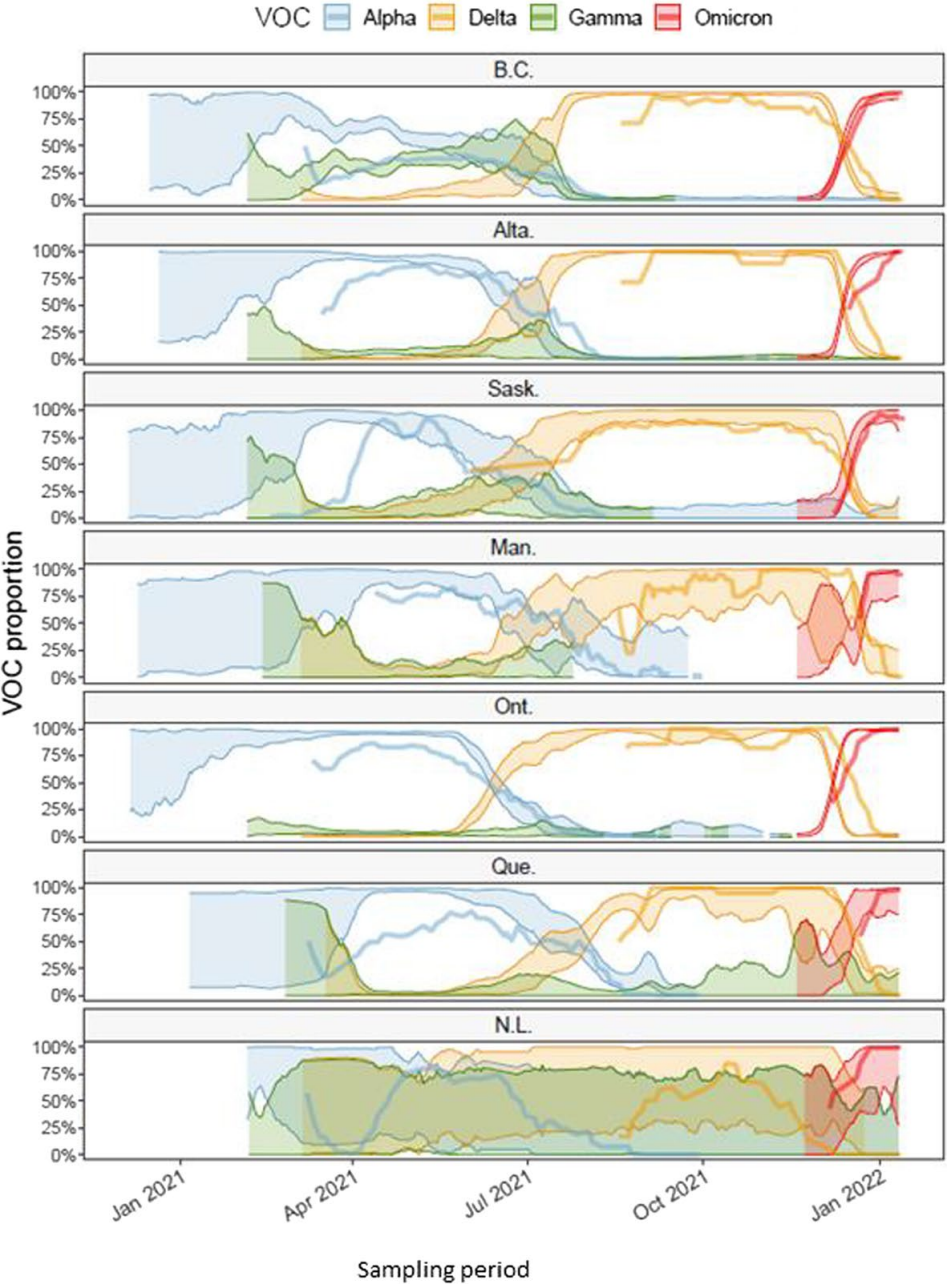
Proportions of VOCs circulating in each province, estimated from the sequencing of clinical samples (shaded area representing the upper and lower 95% confidence interval) and RT-qPCR applied to VOC-defining mutation targets from wastewater samples (thick solid line, representing the mean estimate). Data sources: PHAC (clinical surveillance); PHAC/NML (wastewater surveillance for B.C., Alta., Man., Ont., Que. and N.L); University of Saskatchewan (wastewater surveillance for Sask.). Delta assay was only available after 18-Aug-2021. Abbreviations: British Columbia (B.C.), Alberta (Alta.), Saskatchewan (Sask.), Manitoba (Man.), Ontario (Ont.), Quebec (Que.) and, Newfoundland and Labrador (N.L.)

The trends of VOC proportions observed from the clinical data (shaded areas, Fig. 1) and from the wastewater data (thick lines, Fig. 1) appear similar in all provinces. However, some regional differences were observed. VOC Gamma occurred at a significant frequency only in British Columbia and Saskatchewan, but did not displace the emergence of VOC Delta (Fig. 1). During summer 2021, three VOCs, Alpha, Delta and Gamma, probably co-circulated at substantial levels in Alberta, British Columbia and Saskatchewan, whereas transitions between these VOCs appeared to be faster in Ontario and Quebec. Differences in sizes of clinical samples (Figure S1) affected the size of uncertainties in proportion estimates and result in confidence intervals of different sizes (i.e., confidence intervals broadening when the sample size is small. Figure 1, shaded areas). The proportions of all clinical samples reported that could not be assigned to any VOC, due to screening results being unable to distinguish between circulating VOCs or sequencing results not being classified as a VOC, was variable in time and across provinces (Figure S2). The proportion of unidentified VOCs in the clinical samples was generally below approximately 80% in each province, except during the early phase of the VOC Alpha and after July 2021 in Quebec and Manitoba (Figure S2). Hence, generally there was sufficient sample size to estimate VOC proportions from clinical samples (the uncertainty being reflected in magnitudes of confidence intervals in Fig. 1).

**Fig. 2:**
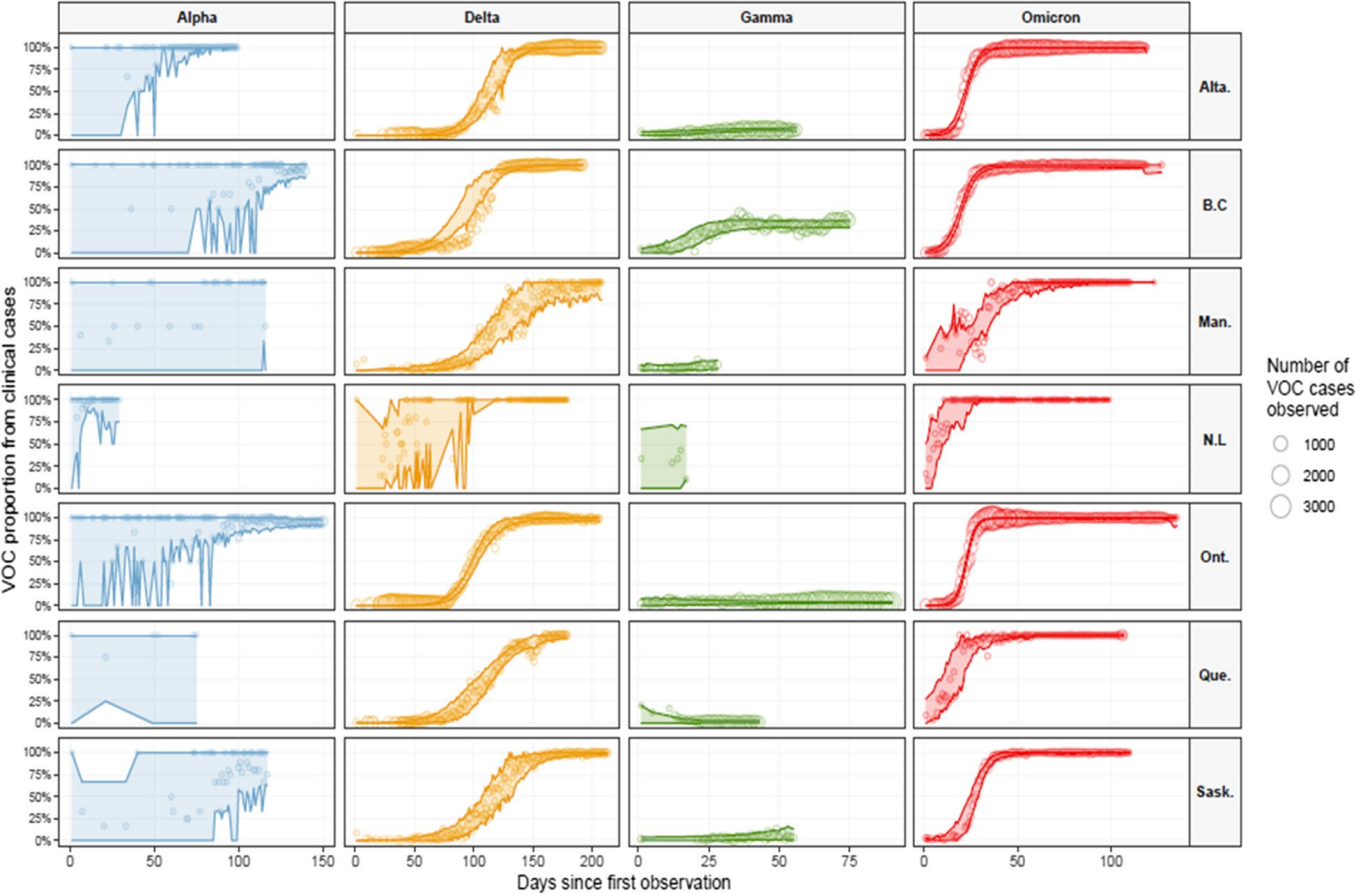
Logistic growth model fit to initial invasion. The circles represent the observed proportion of the VOC; circle size is proportional to the number of clinical samples identified as that VOC. The shaded area and solid lines represent the upper and lower bounds of the 95% credible intervals for prediction of the logistic growth model. Abbreviations: British Columbia (B.C), Alberta (Alta.), Saskatchewan (Sask.), Manitoba (Man.), Ontario (Ont.), Quebec (Que.) and, Newfoundland and Labrador (N.L)

In most provinces, proportions of VOCs measured from wastewater samples (Fig. 1, thick lines) show similar trends as the proportions estimated from clinical surveillance, especially for the Omicron VOC. The assay used for Delta in wastewater by NML became available after August 18, 2021, hence there is no wastewater-based estimates of Delta proportions before this date (except for Saskatoon). Proportions of VOCs observed in municipal wastewater only are shown in Figure S3. The fit of the logistic growth model on clinical data of VOCs observed during their initial introduction phase is shown in Fig. 2. Credible predictive intervals for VOC Alpha are broad because clinical data were collected too late to capture the early invasion stage (Fig. 2). According to the logistic fit for VOC Gamma we infer that this VOC circulated at significant levels only British Columbia. It is important to note that Gamma VOC was not detected in wastewater in any provinces and not even in British Columbia (Figure S3) where Gamma reached 50% proportion (Fig. 2).

### Speed of replacement of VOC proportions

**Fig. 3:**
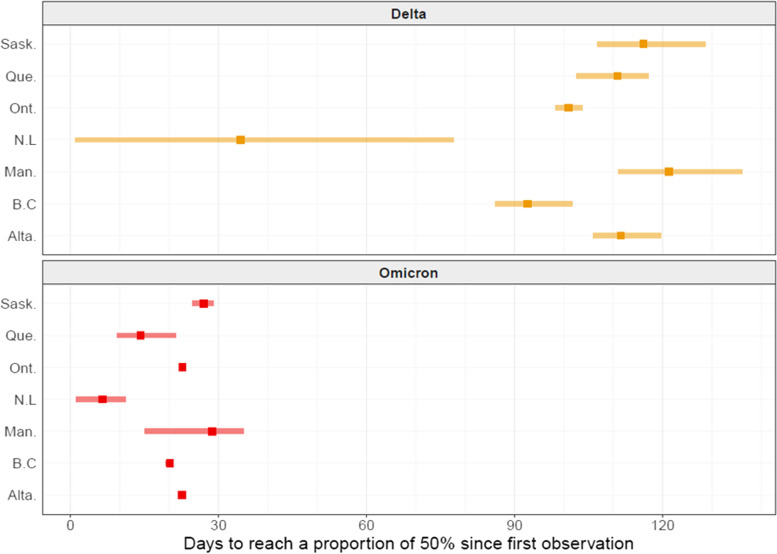
Time taken for each VOC to reach a proportion of 50% after its first observation in the respective province. Point represents the mean estimate, and the width of the horizontal blue segment is the predictive 95% credible interval

Alpha, Delta and Omicron were the only VOCs that successfully invaded all jurisdictions. The uncertainty for VOC Alpha is very large because there is enough data on the initial introduction phase for Delta and Omicron only (Figure S2). The rate of spread for these two VOCs were similar among provinces based on the estimated time it took after the first observation in Canada to reach a proportion of 50% (Fig. 3). The estimate for VOC Delta in Newfoundland is an outlier and probably affected by the small sample size. The maximum number of samples sequenced in newfoundland was less than 1000 (Figure S1). VOC Delta took between three and four months to become the major circulating variant in Canada, but VOC Omicron required less than one month to become dominant. The same results displayed with respect to calendar dates are presented in Figure S4.

**Fig. 4:**
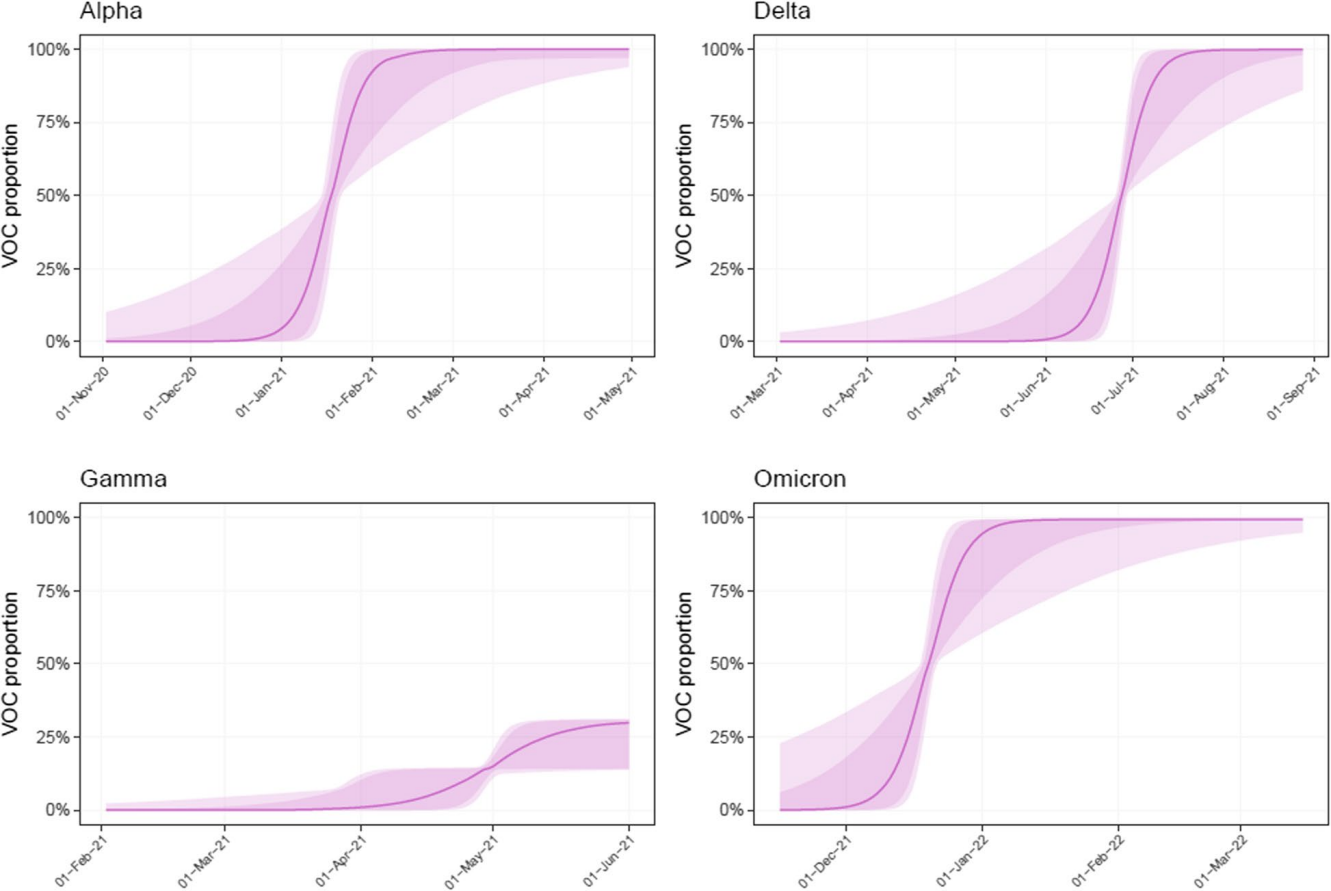
Estimates of circulating VOC proportions in Canada. Thick line represents the mean, dark (resp. light) shaded area the 80% (resp. 95%) credible interval

The fit of the hierarchical model to clinical data provides a national perspective of the initial invasion dynamics for each VOC. Posterior estimates presented in Fig. 4 suggest that Alpha had nearly fully invaded Canada by early January 2021, Delta by early July 2021, and Omicron by early January 2022. Despite its inability to invade fully, Gamma VOC probably reached a maximum proportion of about 25% nationally by early June 2021.

## Discussion

In recent time, COVID-19 pandemic has provided the first opportunity to observe, in detail and near real time, an emerging pathogen spreading in human populations globally. Moreover, due to unprecedented sequencing efforts with over 8 million clinical sequences collected worldwide and uploaded to the sharing platform GISAID (www.gisaid.org) in less than two years (2021 -2022), it has been possible to track the evolution of SARS-CoV-2. This entails keeping an eye out for the appearance of different lineages and outlining their rate of dissemination and level of disease in different populations.

### Geographic Difference of VOC Dynamic Trends across Canada

This study reports the spread of four SARS-CoV-2 variants of concern (VOCs) across seven Canadian provinces by use of clinical and wastewater surveillance data, covering approximately 95% and 25% of the population of Canada, respectively. Despite being a geographically large country, in which public health measures related to COVID-19 were decided and implemented at the provincial level, synchrony in the VOC introduction was observed in all jurisdictions with similar speeds of invasion for each VOC. This is remarkable because there were notable differences in management of COVID-19 by implementation of various public health measures among provinces [29]. Despite having a small sample size (due to its population size) Newfoundland and Labrador appeared to be an outlier. This province had very different public health measures, at least as it pertained to the movement of people, and it managed to keep the number of COVID-19 cases relatively low, at least until the Omicron wave. At the time when Delta started to invade, there were virtually no local transmission in this province. Overall, whereas variations in public health measures among jurisdictions did not appear to have significant effects on the onset or the rate of the transmission of VOCs, they undoubtedly contributed to controlling the time it takes to reach 100% proportion.

The similarity in the emergence of each VOC across Canada might have been caused by simultaneous importation events (e.g., international travel linked to essential businesses and essential services never stopped in Canada) and the intrinsic high contagiousness of SARS-CoV-2 even among asymptomatic cases. Large uncertainties in estimates of proportions of VOCs, which result in broad confidence intervals, underscore the importance of having sufficiently large and robust programs to surveil lineages to accurately monitor VOC emergence and spread. The proportion of clinical samples without VOC identification was variable in our dataset (Figure S2) and not explicitly modelled, hence periods with a low proportion should broaden even more our confidence intervals. Moreover, such a surveillance should rely, partially, on random sampling to avoid biased estimates.

### Application of modeling data to provide policy-making metrics

Effective reproduction numbers (also known as “Rt”), which are critical metrics to inform public health actions but lack the time dimension, have been estimated for VOCs in various Canadian locations [30]. It is possible to estimate Rt using both clinical and wastewater data [31, 32]. Here, estimates based on a logistic growth model were provided as a complementary model for Rt metrics and provide practical, easy-to-understand, quantitative information about the speed of initial spread. Rates of spread reported here provide benchmarks for what to expect from future SARS-CoV-2 VOCs that might occur. VOCs Delta and Gamma, both having few novel mutations, took about four months to become predominant. However, Omicron with multiple novel mutations on the spike protein reached a proportion of 50% in less than a month. Our findings are similar to those of other studies have found in Canada [33–35] and in other countries [11–13, 16, 18, 20].

The results presented here (synchrony and rate of spread of VOCs, similarity of circulating VOCs proportions from wastewater and clinical data) are important metrics that can assist in anticipating the necessary public health decision time should other VOCs emerge in the future. This study uses two data streams: clinical reports of COVID-19 cases (used to fit statistical models) and SARS-CoV-2 concentration in wastewater (for comparison). Since they each have different sources of bias, the approximate agreement between both sources strengthens inferences of dynamics of relative proportions of VOCs in Canada. Clinical sampling is usually not random, including in Canada. Some socio-demographic groups, such as healthcare professionals, might be over-represented. Outbreaks may also be over-represented. Other groups might have limited access to testing and sequencing capacity might be limited during large infection waves, which might exacerbate these and other biases. Clinical testing strategies (including capacity, and targeted populations) also varied by jurisdiction. Wastewater sampling might be affected by environmental events, such as large rainfalls or snowmelts, industrial wastes, and events causing a large influx of people into a sewershed, such as sporting events or conferences. Finally, areas studied were limited to major urban centers and did not reflect the burden of disease in remote regions of Canada.

### WBE and clinical data can be complementary to support comparative estimates of infection disease

The agreement between clinical and wastewater data, especially that observed during the Omicron wave, validates the ability to monitor proportions of VOCs circulating in communities from data collected in wastewater [10, 36, 37]. The WBE estimates can also provide an opportunity to better triangulate VOC proportions in locations where fewer clinical samples have been available, such as Newfoundland and Labrador (Fig. 1), where larger uncertainty from clinical data is indicated by the widths of confidence intervals). It is important to note that the difference in the proportion of clinical VOC and wastewater VOC might be related to the geographical locations covered for wastewater and clinical samples. The clinical surveillance in most cases represent the entire province, while the wastewater is just a representative of the major cities. High positive correlation (r > 0.7) has been established between clinical VOC and wastewater VOC when they both covered the same area [24]. Even if it does not reach the level of precision of clinical surveillance, WBE based on PCR assays targeted on VOC-defining mutations has the potential to be a cost-effective surveillance method that may be more consistent both in time and across jurisdictions (especially when the wastewater analysis is performed using a stable assay in each laboratory in order to perform a longitudinal analysis – as it is the case here in this study). Although currently not timely enough to provide a near real-time surveillance, full sequencing of SARS-CoV-2 genetic materials found in wastewater can complement PCR-based assays because it provides a more complete and detailed picture of circulating VOCs, and can potentially identify new dominant variants (but the development and validation time for new wastewater-based assays may hamper timeliness) [17, 38] There is a clear advantage to perform full sequencing (vs. PCR-based assay) when there are multiple overlapping mutations defining different lineages, as this is the case in the post-Omicron era.

Interpretation of differences between proportions of various VOCs estimated from clinical and wastewater data is not evident. Indeed, a clinical surveillance that indicates a larger proportion of a particular VOC than that determined in WBE estimation, for example Alpha during spring 2021 in Ontario and Quebec, could be caused by a bias where clinical samples were preferentially selected among patients infected with Alpha because they were more symptomatic compared to wild types. Other factors that could bias VOC proportions can be periods of enhanced testing for contacts by some jurisdictions and preferential sequencing for travelers from certain regions. Alternatively, the lesser proportion estimated from WBE might be caused by lesser sensitivity of the laboratory assay. There is also an intrinsic demographic mismatch between clinical surveillance, which is performed province wide, and wastewater samples that may only be collected in only a few large cities in each province (in this study, the single largest city). Hence, differences between proportions of VOCs estimated from clinical and wastewater data can also be caused by a VOC emergence that might not occur at the same time and speed in the largest cities of a province as it does in the whole province. In addition, sequencing of wastewater could be challenging when the concentration is very low. Finally, the small sample size of clinical samples experienced at times in some provinces (Figures S1 and S2) may have impacted the accuracy of our analyses. A mismatch between clinical and wastewater VOC or trend has been associated with reduced clinical testing [39]. Thus, multiple factors might account for mismatched between clinical VOC and wastewater VOC, hence, both should be used to complement each other.

It is important to note that this study has certain limitations. The analysis of wastewater samples was conducted in a different lab from the one where VOC analyses were performed. This means that factors such as dilution, extraction efficiency, nature of assay, and RNA standards could have had an impact on the results. However, it should be noted that the comparison of wastewater results was not conducted between labs, which helps to reduce the effect of this limitation on the study.

## Conclusion

This study presents an analysis of the spread of four SARS-CoV-2 variants of concern (VOCs) (alpha, gamma, delta and Omicron) across seven Canadian provinces using clinical and wastewater surveillance data. Based on clinical surveillance data the SARS-CoV-2 VOCs have synchronously invaded different parts of Canada despite the wide geographic distance and with various intensities of independently implemented COVID-19 public health measures. Wastewater-based surveillance can be a good indicator of VOCs circulation in communities and can complement clinical surveillance. Comparative estimates of the rapidity of spread and synchrony for past waves driven by VOCs can support preparedness for next VOCs and, to some extent, next pandemics from other pathogens. Experience from COVID-19 where there was an enormous amount of information shows that wastewater can be an additional tool that can and should be applied to future emerging pathogens or VOCs of SASR-CoV-2. Application of modelling enhance the interpretations of wastewater results and make it more meaningful.

### Supplementary Information

**Additional file 1. **

## Data Availability

All datasets analysed during the current study are available in supplemental material. If additional information is needed on datasets used and/or analyzed during the current study they are available from the first author (David Chapremdon) on reasonable request.

## Abbreviations

SARS-CoV-2: Severe acute respiratory syndrome coronavirus 2
VOC: Variants of Concern
WBE: Wastewater-based epidemiology
PHAC: Public Health Agency of Canada
NML: National Microbiology Laboratory
US-CDC: United State Centers for Disease Control and Prevention
V: Variant
t: Time
B.C.: British Columbia
Alta.: Alberta
Sask.: Saskatchewan
Man.: Manitoba
Ont.: Ontario
Que.: Quebec
N.L.: Newfoundland and Labrador
